# Use of a cancer registry is preferable to a direct-to-community approach for recruitment to a cohort study of wellbeing in women newly diagnosed with invasive breast cancer

**DOI:** 10.1186/1471-2407-8-126

**Published:** 2008-05-02

**Authors:** Marijana Lijovic, Susan R Davis, Pam Fradkin, Maria La China, Helen Farrugia, Rory Wolfe, Robin J Bell

**Affiliations:** 1 Women's Health Program, Department of Medicine, Monash University, Alfred Hospital, Prahran, Victoria 3181, Australia; 2 Cancer Epidemiology Centre, The Cancer Council Victoria, Carlton, Victoria 3053, Australia; 3 Department of Epidemiology and Preventive Medicine, Monash University, Alfred Hospital, Prahran, Victoria 3181, Australia

## Abstract

**Background:**

Breast cancer (BC) mortality is declining such that the number of survivors of BC in the community is increasing. BC survivors report a range of sequelae from their cancer and its management beyond the period of their immediate treatment. Previous studies to document these have generally been small, clinic-based or commenced years after diagnosis. We have recruited a large cohort of women newly diagnosed with invasive BC from the community who will be followed for five years in order to systematically document the physical, psychological and socio-economic consequences of BC and its treatment. The aim of this manuscript is to describe the issues encountered in the recruitment of this community-based study population.

**Methods:**

Women residing in the southern Australian state of Victoria newly diagnosed with invasive BC were recruited to this cohort study using two approaches: directly from the community using an advertising campaign and contemporaneously using an invitation to participate from the Victorian Cancer Registry (VCR).

**Results:**

Over the two and half year recruitment period, 2135 women were recruited and agreed to receive the enrollment questionnaire (EQ). Of these, 1684 women were eligible and completed an EQ, with the majority of participants having been recruited through the VCR (n = 1321). Only 16% of women contacted by the VCR actively refused participation following a letter of invitation and phone follow-up. The age distribution and tumour characteristics of participants are consistent with state-wide data and their residential postcodes include 400 of a possible 699. Recruitment through a direct community awareness program aimed at women with newly diagnosed invasive BC was difficult, labour-intensive and expensive. Barriers to the recruitment process were identified.

**Conclusion:**

Most of the women in this study were recruited through a state-based cancer registry. Limitations to recruitment occurred because we required questionnaires to be completed within 12 months of diagnosis in a setting where there is several months delay in notification of new cases to the Registry. Characteristics of the cohort suggest that it is generally representative of women in the state of Victoria newly diagnosed with BC.

## Background

Breast cancer (BC) is the most common cancer in women in developed countries and improved survival is leading to an increase in the prevalence of BC survivors in the community. Relative survival data from Victoria shows that the majority (84%) of women diagnosed with BC are alive 5 years after diagnosis and approximately 68% are still alive after 10 years [1]. In Victoria, five year survival of women diagnosed with BC rose by 13% between 1990 and 2004 [2]. The number of people living with cancer in the USA has risen from 1.5% of the population in 1971 to 3.5% in 2001, with 22% of these individuals having had BC [3]. The quality of life of BC survivors is a major public health issue [4,5], yet no large population-based study of quality of life for women recently diagnosed with BC has been reported.

Some studies [5-7] have suggested that BC impacts on health-related quality of life and psychological wellbeing, however these have been small, mainly clinic-based studies which are mostly limited by age group or time since diagnosis. The socioeconomic consequences of BC remain under-researched. For the most part, BC is assumed to impact on family structure, employment and other psycho-social aspects of life. The findings of a review suggest that the belief that women with BC are abandoned by their partners is false [8]. There is some information about work force participation of women with BC [9,10] and there is some Australian data on the economic costs to the individual with BC within the first 18 months after diagnosis [11]. However, much more information about the consequences of BC diagnosis and treatment and the impact on overall quality of life is needed.

In summary, there has been little systematic follow up of women's experiences after treatment for BC outside therapeutic trials. This is important because therapeutic trials always include a selected patient population and thus the findings can never be fully generalized.

This study was designed to systematically document the physical, psychological and socio-economic consequences of BC, longitudinally, amongst women living in the southern Australian state of Victoria recruited within the first year of their diagnosis and then followed annually for 5 years. We are particularly interested in the changes in quality of life experienced by women in the years immediately following their diagnosis and treatment of BC, including the impact of a range of BC therapies (such as the symptoms of estrogen deficiency) as well as the eventual impact of complications such as recurrence.

Our aim was to ensure that recruitment to this 5 year cohort study was community-based, rather than clinic-based, so that the cohort was widely representative of women newly diagnosed with invasive BC. We were also keen to ensure recruitment occurred within the first year after diagnosis so that we could report on the evolution of issues identified as being of importance to BC survivors starting soon after diagnosis and initial treatment. This report documents the challenges associated with recruitment to the cohort study.

## Methods

### Summary of the study methods

This study is being conducted entirely by questionnaire. Participants are asked to complete an enrollment questionnaire (EQ) at baseline and five follow-up questionnaires at intervals 1, 2, 3, 4 and 5 years after the EQ has been returned. Questionnaires are posted to the home address of participants and completed questionnaires are mailed back in a reply-paid envelope. There is no cost to the participant from being involved in the study. The study questionnaires are only provided in English as we do not have the resources to make the questionnaires available in other languages. A free call (1800) telephone number is available to participants if they require assistance with the questionnaires; this service is only available in English. Participation in the study is voluntary and does not affect any aspect of a participant's treatment by their clinician.

A woman was considered a consenting participant of the study when she returned the EQ along with a signed copy of a participant consent form. Each woman is free to withdraw from the study at any time.

### Ethics approval and support from other organisations

The study was approved by the Human Research Ethics Committee of The Cancer Council Victoria and the Standing Committee on Ethics in Research involving Humans of Monash University and recruitment was supported by more than 50 Victorian hospitals and health centres. The conduct of this study also has the support of BreastScreen Victoria (the Victorian component of BreastScreen Australia, an Australian government-funded mammography screening program) and the Victorian Cooperative Oncology Group – Breast Trials Sub-Committee.

### Study advisory group

A study advisory group was established and consists of eight individuals, including four breast surgeons, one medical oncologist, one representative from BreastScreen Victoria, one representative from The Cancer Council Victoria and one BC consumer advocate. This group has ongoing input into the design and execution of the study.

### Study eligibility criteria

Ductal and lobular carcinomas account for more than 95% of BC cases. Women with these types of BC were eligible to take part in the study. Other study eligibility criteria are outlined in Table 1. For this study, any of the following breast conditions in the absence of a diagnosis of primary invasive BC rendered a woman ineligible to take part in the study: Ductal carcinoma in situ (DCIS), Phyllodes tumour, Paget's Disease, Lymphoma and Sarcoma.

**Table 1:** Study eligibility criteria.

• Date of first diagnosis of invasive breast cancer satisfies both of the following conditions:
○ After 1 June 2004
○ Not more than 12 months before the time of study enrollment
• 18 years of age or older
• Victorian residential address
• Histological confirmation of primary invasive breast cancer excluding: Ductal carcinoma in situ (DCIS), Phyllodes tumour, Paget's Disease, Lymphoma and Sarcoma
• Good comprehension of English language

### Sample size considerations

The primary aim of this study is to document self-reported wellbeing in BC survivors and to use linear regression to model what factors are contributing to variation in wellbeing. For this analysis it is desirable that the number of observations is at least 10 times the number of variables to be included in the model [12]. With 1684 women in the cohort, the sample size will be adequate to investigate wellbeing in subgroups of women and to include a range of variables related to the cancer and its treatment, physical symptoms and socio-economic measures.

### Recruitment strategies

Women were recruited concurrently by two methods in order to maximize recruitment.

The community-based approach involved doctors, breast care nurses, clinics and a public awareness campaign, such that women interested in joining the study were encouraged to contact our centre (Women's Health Program, or WHP) directly.

All major metropolitan and rural hospitals and health centres in Victoria were approached to be involved in recruitment. These centres were identified from lists provided by The Department of Human Services (DHS) [13-15]. The hospital and health centre lists, which included both private and public health services, were accessed in July 2004.

Telephone numbers provided in these lists were used to make initial contact with each centre to ascertain if there was a breast clinic (surgery or oncology) at the site. If there was a breast surgical or oncology clinic in the centre, permission was sought for study brochures and promotional posters to be displayed in the clinic and for relevant clinic personnel to make the study known to eligible women. Written approval from the centre was required before study material could be displayed. Of 113 Victorian public metropolitan and rural hospitals and health centres, 25 metropolitan and 31 rural centres did not have a breast surgical or oncology clinic. Of the remaining 57 centres, approval for promotion of the study was received from the hospital ethics committee (or senior nursing management, if it was a small site) from 49 centres. This followed submission of documentation outlining prior ethics approval for the conduct of the study from The Cancer Council Victoria, along with other relevant study documentation. One centre did not agree to the promotion of the study and seven centres did not respond to repeated requests about our study. At participating centres, women were provided with a study brochure and asked to sign and return a 'consent for future contact' form by post (prepaid card). Only women who registered their interest in the study were sent a questionnaire.

Women could also register their interest in the study online by visiting the study website [16]. During the period of study recruitment, a number of promotions were used in order to increase the profile of the study. These activities included the display of posters in doctors' rooms, blood collection centres, sports clubs and on public transport (trams), articles in newspapers and magazines, free bookmarks distributed through bookstores and presentations at conferences and forums related to BC.

Recruitment via the Victorian Cancer Registry (VCR) involved letters of invitation issued directly from the VCR to women with newly-notified, invasive BC. It is a statutory requirement that bodies such as hospitals, pathology laboratories and BreastScreen Victoria notify the VCR of new cases of BC. Thus notification of a single case of invasive BC may come from multiple sources. The definitive diagnostic information, however, is obtained from the pathology report. The pathology report refers either to a surgical specimen or a core biopsy specimen.

The recruitment process via the VCR was initiated for women who had not already been recruited directly to the study. On a monthly basis, the WHP provided the VCR with the names of women who had already been recruited to the study directly from the community. This was done to ensure that any woman who had directly registered interest in the study was not inadvertently contacted again by the VCR.

**Figure 1:**
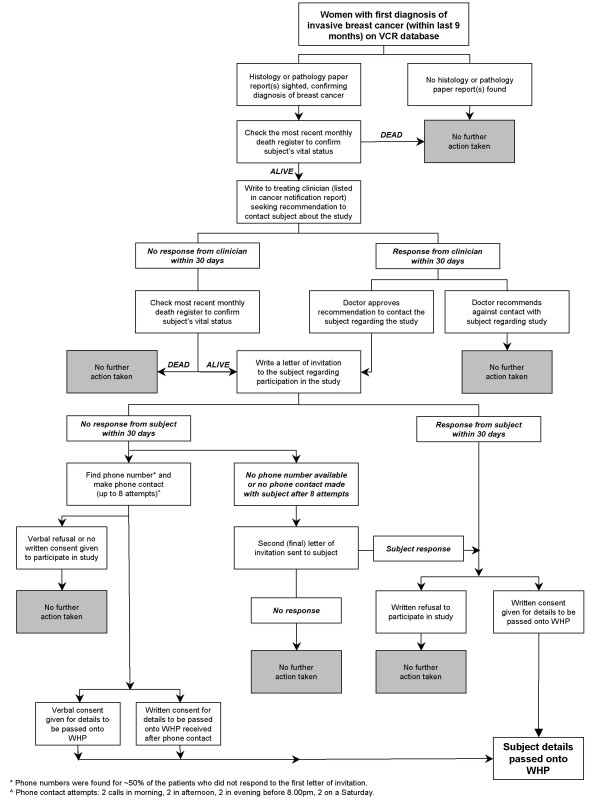
Process of recruitment through the Victorian Cancer Registry (VCR).

The recruitment of participants via the VCR was a labour-intensive process that involved a number of stages (see Figure 1). Throughout the recruitment pathway, monthly death checks were performed to avoid initiating contact with the doctor or family of a recently deceased woman. Briefly, the first step was to write to the treating clinician for advice aimed to minimise inappropriate contacts, such as other co-morbidities (e.g. dementia), difficult family circumstances or limited English comprehension skills which would contraindicate participation. If, within 30 days, the clinician had not responded, a letter of invitation seeking consent for their contact details to be released to the WHP was sent directly to the woman. Women who did not respond to the initial invitation letter from the VCR were followed up via a series of phone calls from the VCR and, if no phone contact was possible, a second letter. In order for women to complete their EQ within 12 months of the date of their diagnosis, recruitment of women through the VCR had to allow for the time required for the process shown in Figure 1. The process of attempting contact with the treating clinician and the eligible woman could take up to 2 months. In order to ensure that an EQ was completed within 12 months of diagnosis, recruitment of a particular woman via the VCR had to commence within 9 months of the diagnosis of BC.

### Completion of enrollment questionnaires (EQs)

Immediately after a woman registered an expression of interest to participate, she was posted an EQ and participant consent and information form. Written consent was obtained from each eligible participant.

## Results

### Study population

In total, 2135 women agreed to participate in the study and to receive the EQ. Of these women, 388 did not return a completed EQ. The remaining 1747 women returned a completed EQ, giving a questionnaire response rate of just over 80% (1747/2135). This response rate is a result of both spontaneous return of the questionnaire and return of the questionnaire after the participant received a single reminder letter 3 months after the EQ was posted. The 1747 completed questionnaires included 63 questionnaires from women considered ineligible for our study. These questionnaires were almost all (90%) from women who had been recruited directly through the WHP for whom, after exchanging information with the VCR, it was established that they did not meet the eligibility criteria. Of these 63 women, 28 were found to have DCIS rather than invasive BC, 14 had been diagnosed more than 12 months earlier, 14 had had an invasive BC diagnosed on a previous occasion, 2 were diagnosed outside of the state of Victoria and 5 were excluded on the basis of their tumour histology. Altogether, 1684 eligible women completed an EQ; of these, 363 had been recruited directly through the WHP and the remainder, 1321, through the VCR.

We have estimated that about 7870 women would have been newly diagnosed with invasive BC in the state of Victoria during the recruitment period of 31 months between June 2004 and December 2006 [17]. However, due to the combination of eligibility requirements (Table 1) that women had to be diagnosed after June 2004 and had to complete the questionnaire within 12 months of diagnosis, combined with the average delay between diagnosis and notification to the cancer registry being about 5 months (personal communication-HF), meant that a much smaller number than 7870 women truly represented the denominator for our study. We did not recruit women notified to the Registry in the second half of 2004 who were diagnosed prior to June 2004 and we would not have had available to us women diagnosed in the second half of 2006 who were notified to the Registry after December 31, 2006. At the peak of our recruitment (2006 – when about 3000 new cases would have been diagnosed), doctors were approached about 1816 women, 1620 women were approached directly and 1032 consented to participate. The treating clinicians recommended against 5% of the identified BC cases being approached for recruitment and 1% of potential recruits had died. Only 16% of women who were contacted by the VCR actively refused participation.

The women were recruited from 400 out of 699 postcodes around Victoria. According to geographical classifications of the participants' postcodes, 68% of the study participants lived in metropolitan Victoria, whilst 32% lived in country Victoria. This is in line with recent Australian data [18] which show that 73% of adult females (18 years of age and over) live in Metropolitan Victoria, whilst 27% live in country Victoria. The age distribution of women recruited to this study is very similar to that of all women with invasive BC registered with the VCR [17], except for an under-representation of elderly women (Table 2). The distribution of tumour size in the women in our cohort was very similar to what has been published for the state population of women newly diagnosed with invasive BC [19] (Table 2).

**Table 2:** Age and tumour size distribution of the study participants compared with Victorian women diagnosed with breast cancer.

**Age group**	**Victorian women diagnosed with invasive breast cancer in 2004 [17]**	**Women in 'Health and Wellbeing after Breast Cancer Study'**
<40 years	5.6%	6.1%
40–59 years	46.2%	54.3%
60–79 years	37.0%	36.2%
80+ years	11.2%	3.4%

**Tumour size **(where known)	**Victorian women diagnosed with invasive breast cancer in 2000 [19]**	**Women in 'Health and Wellbeing after Breast Cancer Study'**

<1 cm	19.1%	17.9%
1-<2 cm	42.4%	44.0%
2-<5 cm	34.1%	35.0%
5-<10 cm	3.9%	2.9%
10+ cm	0.5%	0.2%

## Discussion

This report describes the obstacles encountered in recruiting women newly diagnosed with invasive BC to a longitudinal study. Firstly, recruitment through the state cancer registry was, at face value, more successful than a system of recruitment directly through the community, although (as discussed below) these methods of recruitment may have interacted with each other. Different methods of recruitment to research studies have been analysed, including the use of tumour registries for the study of cancer survivors [20]. Barriers to participation have been identified, as well as effective and less effective sources of study participants. There is evidence that using more than one recruitment method is advantageous [21,22]. Overall, methods such as direct mailings are considered successful but relatively expensive, whereas physician or clinic referral and media advertising (used in our WHP strategy) are considered to be less expensive but have a lower yield than direct methods [21,23].

The primary factor impeding recruitment through the cancer registry was the combination of our requirement of completion of the enrollment questionnaire within 12 months of diagnosis in the setting of delayed notification to the Registry. The fact that the cohort appears to be largely representative of all women newly diagnosed with invasive BC in Victoria suggests that the barriers to recruitment did not produce sampling bias.

Our initial assessment was that recruitment directly from the community was disappointing, most likely because it required busy physicians to be proactive in facilitating recruitment and women to be proactive in joining a research study at a time when their BC treatment was their first priority. Recruitment via the VCR was more successful. Although we were recruiting to this study at a time when the VCR was under extraordinary pressure due to a change in computer software, where eligible women were invited to participate by the VCR, the relatively low active refusal rate may reflect the success of the public awareness campaign. Thus, when women were directly approached by the VCR, we speculate that they may have already been exposed to the concept of the study in the community.

Recruitment via the VCR required the employment of a full-time data manager dedicated to recruitment for this project for more than 2 years. The crude cost of recruiting each consenting participant via the VCR was approximately $100, determined by dividing the data manager's salary over the period of recruitment by the total number of women recruited using this method. Considering the time dedicated to the community recruitment approach by the study co-ordinator and senior research staff within the WHP (without considering time contributions of breast care nurses working in clinics and hospitals talking to women about the study), the cost per woman recruited by this method was probably even greater. The combination of cost, difficulty of defining an appropriate denominator and the relatively low recruitment rate argue against the "direct from the community" method of recruitment in the establishment of cohorts such as the one described in this manuscript.

We are aware of some biases acting within our recruitment process. There will be an under-representation of women whose first language is not English. There is likely to be bias favouring the recruitment of women managed in the private rather than the public sector because, for women who had surgery in a public hospital, it may not have been possible for the VCR to identify a managing clinician. There will also be an under-representation of women with advanced disease because managing clinicians may have advised against them being contacted. Anecdotally, older women have been more likely to request assistance with completing the questionnaire, so we were not surprised that elderly women were less interested in volunteering for the study. DCIS is an important part of the total picture of BC, however we made the deliberate decision to limit this study to women with invasive BC as our study would not have been powered for the modelling of wellbeing within the small sub-group of women with DCIS.

Although the recruitment delay through the VCR reduced the number of women approached, there is no reason to believe that there was any bias involved in who was invited to participate and who was not. Our cohort includes women who live in many different parts of Victoria, including rural and remote areas. Women living outside metropolitan and regional centres are frequently under-represented in research studies.

Despite all of these challenges, we finally recruited 1684 Victorian women to a cohort of women newly diagnosed with invasive BC using a population-based approach. With a high level of retention, this number of participants will be adequate for modelling the determinants of wellbeing in women living with BC.

## Conclusion

Our aim was to recruit a cohort of women with minimal selection bias. Recruiting through the state-based cancer registry was the more successful way to do this.

A population-based cohort of women newly diagnosed with invasive BC is difficult and expensive to recruit. However, our cohort of 1684 women is now the largest "population-based" study of wellbeing in BC survivors.

## Abbreviations

BC: breast cancer; EQ: enrollment questionnaire; VCR: Victorian Cancer Registry; WHP: Women's Health Program.

## Competing interests

The authors declare that they have no competing interests.

## Authors' contributions

ML, SRD, PF, MLC, HF and RJB contributed to the design of this study and were involved in the recruitment process. ML, PF and MLC managed the participant database and responded to participant enquiries. RW provided statistical advice regarding sample size and data analysis. All authors drafted, read and approved the final version of this manuscript.

## Pre-publication history

The pre-publication history for this paper can be accessed here:


